# Functional Characterization of the *Bari1* Transposition System

**DOI:** 10.1371/journal.pone.0079385

**Published:** 2013-11-14

**Authors:** Antonio Palazzo, Simona Marconi, Valeria Specchia, Maria Pia Bozzetti, Zoltán Ivics, Ruggiero Caizzi, René Massimiliano Marsano

**Affiliations:** 1 Dipartimento di Biologia, Università di Bari, Bari, Italy; 2 Dipartimento di Scienze e Tecnologie Biologiche ed Ambientali (DiSTeBA), Università del Salento, Lecce, Italy; 3 Division of Medical Biotechnology, Paul Ehrlich Institute, Langen, Germany; Alexander Fleming Biomedical Sciences Research Center, Greece

## Abstract

The transposons of the *Bari* family are mobile genetic elements widespread in the *Drosophila* genus. However, despite a broad diffusion, virtually no information is available on the mechanisms underlying their mobility. In this paper we report the functional characterization of the *Bari* elements transposition system. Using the *Bari1* element as a model, we investigated the subcellular localization of the transposase, its physical interaction with the transposon, and its catalytic activity. The *Bari1* transposase localized in the nucleus and interacted with the terminal sequences of the transposon both *in vitro* and *in vivo*, however, no transposition activity was detected in transposition assays. Profiling of mRNAs expressed by the transposase gene revealed the expression of abnormal, internally processed transposase transcripts encoding truncated, catalytically inactive transposase polypeptides. We hypothesize that a post-transcriptional control mechanism produces transposase-derived polypeptides that effectively repress transposition. Our findings suggest further clues towards understanding the mechanisms that control transposition of an important class of mobile elements, which are both an endogenous source of genomic variability and widely used as transformation vectors/biotechnological tools.

## Introduction

DNA-based transposable elements, also referred as terminal-inverted-repeat elements, Class II transposable elements or transposons *sensu strictu*, use a DNA-mediated mode of transposition, that sets them apart from retrotransposons and retroposons that move via RNA intermediates. Cut-and-paste DNA transposons are grouped into at least 17 superfamilies [1], and rely on a self-encoded transposase to catalyze the transposition reaction.

The transposable elements related to the *Tc1* and *mariner* families (*MLE*) (*IS630-Tc1-mariner* or ItmDx[D/E superfamily] [2] constitute the largest group of cut- and-paste Class II transposons. They are mobile elements of up to 2 Kbp in size, able to move within eukaryotic genomes, and in some cases can constitute high proportions of the genomes they inhabit. These elements usually contain a single, intron-less transposase-encoding gene, typically flanked by two short terminal inverted repeats (TIRs) 19 to 40 bp in length. This relatively simple design combined to a self-encoded transposase able to catalyze all the transposition steps *in vitro* [3] make the transposition of *Tc1/mariner* like elements [4] independent of host factors and could explain why these elements have a such widespread occurrence. In fact *Tc1* elements require just two TIRs separated by a DNA fragment, a transposase source, and magnesium cations as cofactors for transposition [5] [6].

The *Bari* elements belong to the IR-DR group of the *Tc1* lineage, comprising elements with TIRs ranging from 200 to 250 bp in length. This group also includes other *Drosophila* elements such as *S* [7], *Minos* [8], and *Paris* [9], and the reconstructed fish transposon *Sleeping Beauty (SB)* [10], which encode transposases containing a functional bipartite nuclear localization signal (NLS_BP), two HTH motifs in the N-terminal region and an acidic DD34E triad in the C-terminal region [11] [12] [13]. However, the protein motifs of the transposase encoded by *Bari*-like elements have not been functionally characterized. The TIRs of these elements possess three direct repeats (DRs) called the outer DR (ODR), the middle DR (MDR) and the inner DR (IDR), that are the putative binding sites for the transposase and are necessary for the transposition of autonomous elements [14] [15] [13]. The presence of three DRs is not uncommon; in fact, sequence comparison of TIRs belonging to four *Tc1*-like elements (*Sleeping Beauty*, *Paris*, *S*, and *Minos*) has revealed a third conserved DR between ODR and IDR [16] [13].

Although three related *Bari* subfamilies (*Bari1*, *Bari2* and *Bari3*) differing in structural organization and potential transposition autonomy are known to exist in different *Drosophila* species [16], [17], most of the information about the transposition activity of these elements is limited to *Bari1* elements in *D. melanogaster*. *Bari1* elements were found arranged in an 80-copies array in the heterochromatic h39 region of the second chromosome of *D. melanogaster* [18] [19] [20], and highly polymorphic among 90 different populations of *D. melanogaster* analyzed by *in situ* hybridization on polytene chromosomes of salivary glands [21].

Transposition events were identified by molecular analysis in *Drosophila* laboratory stocks showing genomic instability [22] and in the progeny of a single female collected in the wild [23]. Recently, *Bari1* transposition has been observed in a genetic background in which the piRNA pathway, which suppresses transposable elements activity, was deregulated [24].

Several mechanisms regulating transposition rate are well characterized. They are either based on repressors produced by the transposon [25] or are evolved by the host to protect its genome from excessive insertional mutation events [26] [27] [28].

Several DNA transposons can be used as vectors for moving exogenous DNA sequences into chromosomes by mimicking the natural process of horizontal gene transfer under laboratory conditions; these include the plant transposons *Ac/Ds* and *Spm* systems [29] and transposons from animals including *SB* [10] and *piggyBac* [30]. Engineered transposons are useful tools for biotechnology [31], medicine [32] [33] and genetics [34] [35].

To test the essential biological features of the *Bari* family of mobile elements, we utilized the *Bari1* element as model. Here we show that *Bari1* encodes a transposase that compartmentalizes to the nucleus both in insect and mammalian cells, and that it can bind the TIRs of the *Bari1* transposon. Although we were unable to demonstrate its ability to catalyze the transposition reaction, transcriptional analyses identified unexpected transposase transcripts expressed under different experimental conditions. We discuss the possible causes of the low transposition activity of the *Bari1* element *in vivo* and *in vitro*, and propose that, in addition to already known regulatory circuits, a post-transcriptional regulation mechanism may also control the transposition of the *Bari* elements. Due to the relationship of *Bari1* to other well-known mobile elements, these results could be of importance in the field of the transposon biology. Their importance as source of variability in the eukaryotic genome as well as substrates in the development of novel integration tools requires a deep biological knowledge of their regulation repertoire.

## Results

### *In silico* Analysis of the *Bari1* Transposase

**Figure 1:**
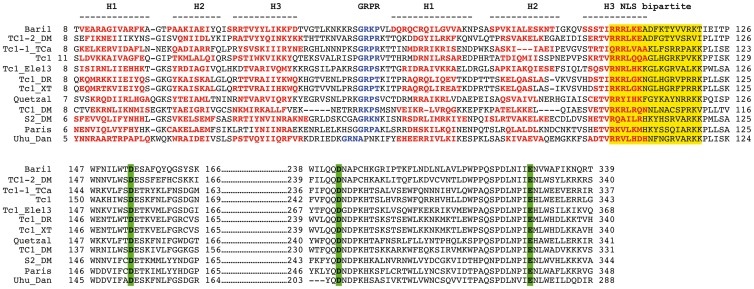
Partial multiple alignment of Tc1-like transposase sequences. Residues of the DNA binding domain (consisting of the H1-H3 alpha helices) are red boldfaced, the GRPR domain is blue boldfaced. NLSs are highlighted in yellow and the acidic triads of the catalytic domains (DDE) are highlighted in green.

To obtain preliminary information on the functional domains of the *Bari1* transposase we compared it with several transposases encoded by Tc1-like elements. Multiple alignment analysis, coupled with secondary structure prediction, identified several functional domains typical of the *Tc1/mariner* transposases (figure 1). Several protein motifs, such as the DNA binding domain, the NLS (Nuclear Localization Signal) and the catalytic domain, have been shown to be of great importance for the transposase function (see [36] for a review), and they can also be recognized in the *Bari1* transposase. A bipartite DNA binding domain thought to be responsible for recognition of the transposon termini can be easily detected at the N-terminus of the protein. This domain is divergent in sequences among the transposases, but the predicted alpha helices of both HTH motifs rely in similar position with respect to each other, indicating functional, rather than sequence conservation.

A slightly divergent GRPR motif (GRKP) motif characteristic of the homeo-domain proteins [37] is also present at position 59 of the *Bari1* transposase and between the two HTH motifs. This domain, followed by an additional HTH region (i.e. the Homeo-like domain), is present in all the transposases aligned. The multiple alignment also highlights the presence of a putative bipartite NLS rich in basic aminoacids. The catalytic domain, characterized by the typical DDE motif, is also recognizable in the primary sequence of *Bari1* transposase.

Notwithstanding these similarities, the PredictProtein prediction tool (http://www.predictprotein.org/) did not predict the NLS signal, while the PSORT prediction tools give ambiguous results (not shown). For these reasons we aimed at experimentally testing nuclear import of the *Bari1* transposase.

### *In vivo* Analysis of the Subcellular Localization of the *Bari1* Transposase

To investigate the nuclear import signals in the *Bari1* transposase we performed a series of immuno-detection tests after overexpression of the transposase in two model cellular systems, the *Drosophila* S2R+ and the human HepG2 cells. After expression of either full length (ASE1) or truncated versions (ASE1/**Δ** 159–339 and ASE1/**Δ** 1–158) of the *Bari1* transposase fused to the V5 tag in the two cell types, the localization of the fusion proteins was detected using a monoclonal anti-V5 antibody.

**Figure 2:**
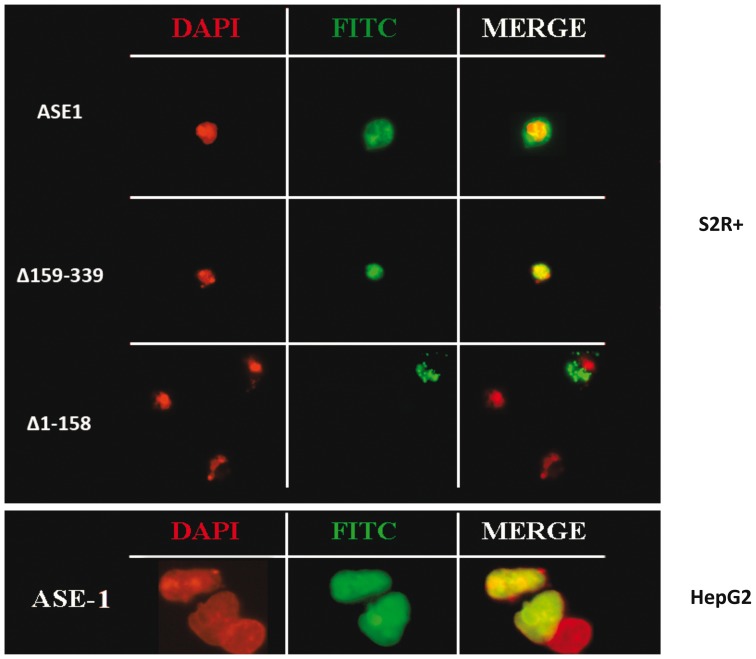
Sub-cellular localization of the *Bari1* transposase. Upper Panel. Localization of the full length (ASE1), the N-terminal (**Δ**159–339) and the carboxyl terminal (**Δ**1–158) portion of the *Bari1* transposase in S2R+ cells. Lower Panel. Localization of the full length *Bari1* transposase in HepG2 cells. The left column shows the DAPI fluorescence signal (nuclear DNA), the middle column shows the FITC fluorescence signal (indicating fusion protein localization), and the right column shows the merged fluorescence signal.

The results obtained are summarized in figure 2 (upper panel, first row), and clearly show that the full-length *Bari1* transposase localizes to the nucleus in both cell types, thus indicating that a nuclear import signal in the protein is functional in both insect and mammalian cells. Furthermore, to precisely map the NLS, we tested the subcellular localization of two truncated forms of *Bari1* transposase in Drosophila cells. As shown in figure 2 (upper panel, second and third row), the ASE1/**Δ** 159–339 amino-terminal portion of *Bari1* transposase (aminoacids 1–158) retains its nuclear localization, while the **Δ**1–158 carboxyl-terminal part (aminoacids 159–339) does not. These results clearly indicate that the first 158 aminoacids of the *Bari1* transposase contain the NLS, which probably maps to the K/R rich amino acidic sequence, a motif conserved in different *Tc1* like transposases (see figure 1).

### *In vitro* Assay of the Binding of the *Bari1* Transposase to the *Bari1* TIRs

In the nucleus, a Tc1-like transposase must bind specifically to the recognition sequence in the transposon DNA (i.e. its terminal inverted repeats). The DNA-binding domains of several Tc1-like transposase, e.g. *Tc1* [38] and *SB* [10], maps to the N-terminal regions, and can be easily predicted using the available secondary structure prediction tools in combination with a multiple alignments (figure 1).

The *Bari1* terminal inverted repeat structure contains three DRs, which are the putative binding sites for the transposase [16]. Here we report the results of extensive *in vitro* and *in vivo* assays of the transposase/transposon interaction.

Expression of the full-length *Bari1* transposase was induced in *E. coli* and the expected 43 kD 6XHis-transposase fusion protein (hereafter T16) was purified by exchange chromatography. Similarly, a 27 kD polypeptide corresponding to the 198 C-terminal aminoacids of the transposase fused to a His-tag (hereafter C9) was expressed and purified (see Figure S1 for detailed figures related to protein expression and purification).

**Figure 3:**
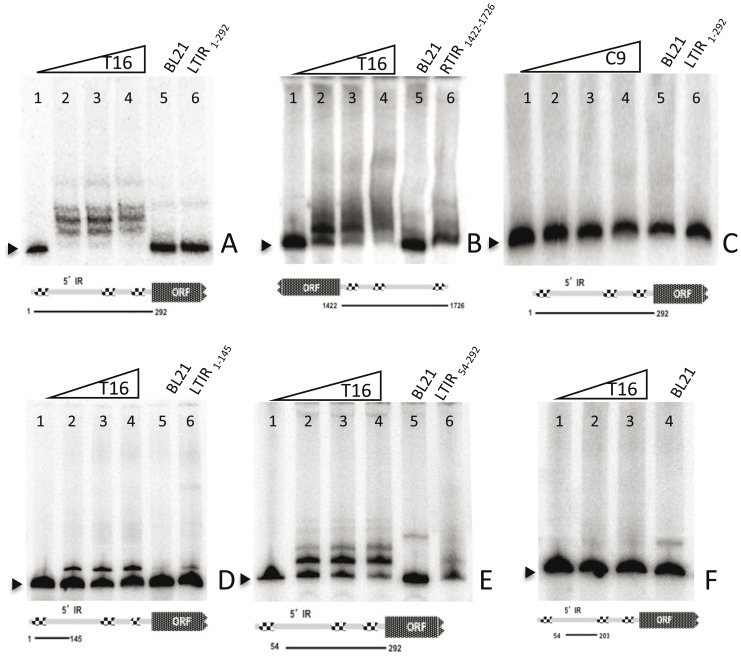
EMSA experiments. Panel A. The radiolabeled fragment corresponding to the full length left TIR containing 3(lane 1) migrates faster than the same fragment incubated with increasing amounts of *Bari1* transposase (T16) (lanes 2, 3, 4). No retardation is observed when the same fragment is incubated with non-induced bacterial extracts (lane 5). The binding is specifically disrupted by a 50X excess of the unlabeled fragment (lane 6). Panel B. Same as panel A, except that the right IR was tested. Panel C. Same as panel A, except that the carboxyl terminal part of the transposase was tested. Panels D, E, F. demonstration of the interaction of the full-length transposase (T16) with left TIR sub-fragments containing one (panel D), two (panel E) or none (panel F) DRs.

The purified proteins obtained were used to assess interaction of the transposase with the *Bari1* terminal sequences in EMSA experiments. As shown in figure 3, DNA fragments containing combinations of the three DRs identified within the *Bari1* left IR [16], were used as target for the binding.

Nucleoprotein complexes were analyzed by EMSA after incubation of the T16 protein with radiolabeled DNA fragments corresponding either to the left or the right IRs of *Bari1*, comparing the patterns observed to those obtained with protein extracts from control (non-induced) bacterial cultures. For both the left and the right IR, slow migrating bands can be observed in the electrophoretic pattern (figure 3, panel A and B, lanes 2–4) compared to the pattern of the free fragment (figure 3, panel A and B, lanes 1), or to the pattern of fragments incubated with protein extracts lacking T16 protein (figure 3, panel A and B, lanes 5). Especially for the left IR, several protein/DNA complexes were visible in the gel, indicating the presence of multiple binding sites for the transposase in the IR, or binding of multiple transposase molecules per IR (figure 3 panel A, lanes 2–4).

Unlabeled IR fragment added in excess as specific competitor DNA inhibited the DNA-protein interaction (figure 3, panels A–E, lanes 6), whereas excess of lambda DNA did not appreciably disrupt the binding (not shown). These results clearly show that the *Bari1* transposase specifically binds both *Bari1* IRs, and so must contain a functional DNA binding domain. Similarly, it can be concluded that the *Bari1* termini contain multiple binding sites for the transposase.

Similar experiments were performed with a purified protein containing the 198 C-terminal aminoacids of the transposase lacking the putative DNA binding domain. As can be observed in figure 3 (panel C) this protein completely lacks the DNA binding properties, as shown by the absence of the slow migrating bands in the electrophoretic pattern. Our results indicate that the transposase domain involved in DNA binding is contained in the first 197 aminoacids of the protein, presumably in the region containing the predicted HTH domains (see figure 1).

Finally, to test the binding properties of the transposase to each of the three DRs of the *Bari1* terminal repeats we performed mobility shift assays using truncated versions of the left terminal repeat containing respectively one (figure 3, panel D), two (figure 3, panel E) or none (figure 3, panel F) of the DRs. The results indicate that the *Bari1* IRs contain multiple binding sites for the transposase, whereas no shifted bands can be observed using a fragment not containing any DR sequence (figure 3, panel F).

### *In vivo* Assay of the Binding of the *Bari1* Transposase to the *Bari1* TIRs

**Figure 4:**
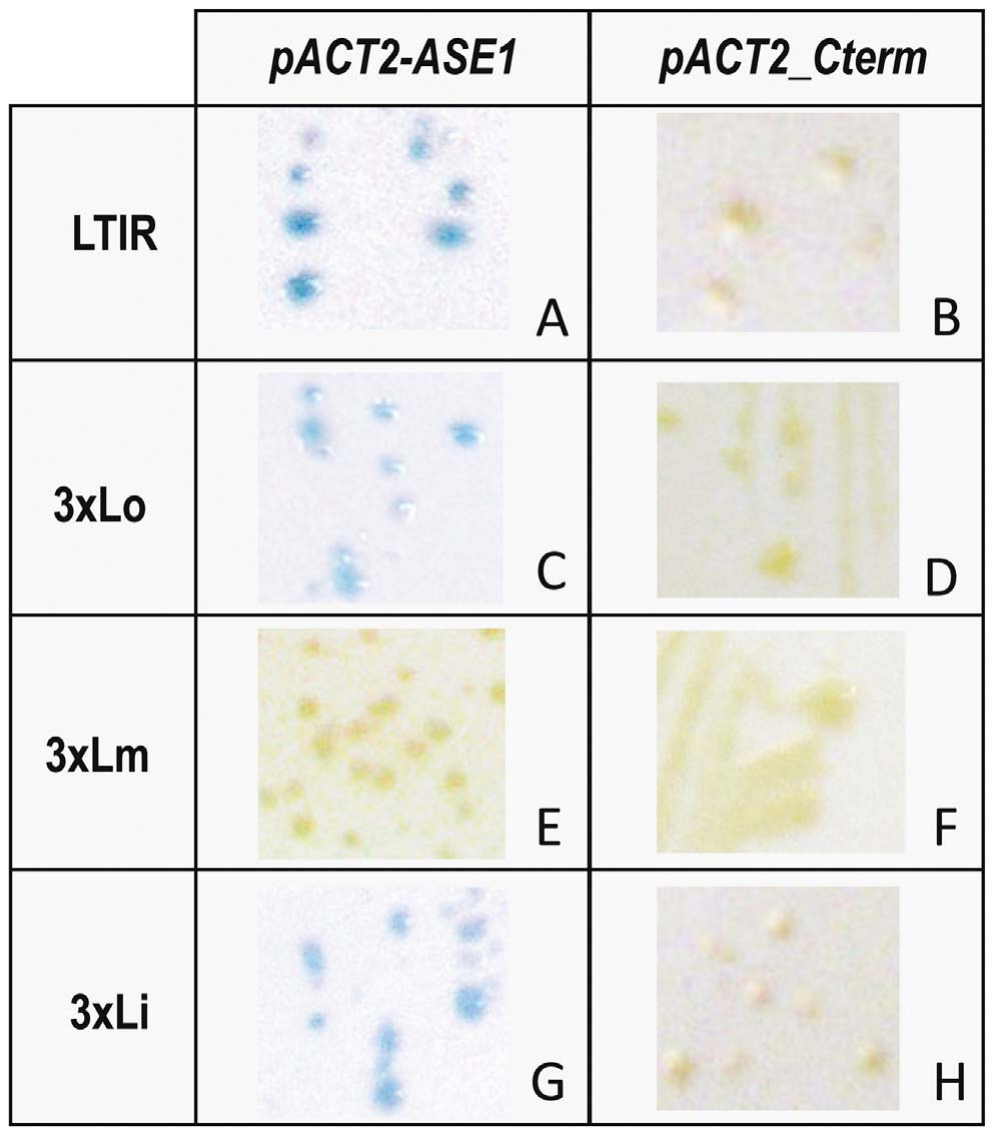
Yeast One hybrid Assay. Left column: LacZ reporter system activation in yeast by full-length transposase expressed due to the interaction with the entire 5′TIR (A), the outer DR (three repeats) (C), the middle DR (three repeats) (E) or the inner DR (three repeats) (G). Right column: *Bari1* transposase lacking the DNA binding domain completely fails to interact with the *Bari1* 5′ TIR or with its DRs (B, D, F, H).

To further demonstrate the DRs-mediated interaction observed in the EMSA experiments, we performed a biased One Hybrid assay in yeast. We constructed yeast strains carrying three copies of either the outer (3×Lo), the middle (3×Lm), or the inner (3×Li) DR, in each case integrated upstream of a LacZ reporter cassette. The three copies of the DRs serve as “bait” for the binding of the transposase “prey” protein expressed from the yeast plasmid pACT2-ASE1 (see Materials and Methods). The LacZ reporter activation is expected only upon bait/prey interaction. Figure 4 shows the results of the **β**-galactosidase assay on integrant yeast strains transformed with a plasmid expressing either the full length (figure 4, panels A, C, E, G) or the carboxyl terminal part (figure 4, panels B, D, F, H) of the transposase. As can be observed in figure 4A, the full-length transposase is able to turn on the LacZ reporter as the expected result of its interaction with the complete left *Bari1* TIR, thus confirming the results of the EMSA experiments (figure 3). Similarly, the LacZ reporter system is also activated in strains transiently expressing the full-length transposase and containing three copies of either the Lo or the Li DR fragments (figure 4, C and G respectively), again confirming that the transposase interacts specifically with the DRs of the terminal inverted repeats. The interaction of the transposase with three copies of the middle DR (Lm) appears to be very weak, as indicated by the very faint blue staining compared to those obtained with the Lo and Li targets. By contrast, and as expected, the carboxyl terminal part of the transposase (aminoacids 142–339), which lacks the DNA binding domain, is completely unable to activate the LacZ reporter when either the entire TIR or the DRs were used as bait (figure 4, B, D, F, H).

Taken together, these results clearly shows that the *Bari1* transposase is able to bind *in vitro* and *in vivo* the DRs contained in the transposon termini, a crucial initial step of the transposition process.

### Excision and Integration are Rate-limiting Steps of *Bari1* Transposition

**Figure 5:**
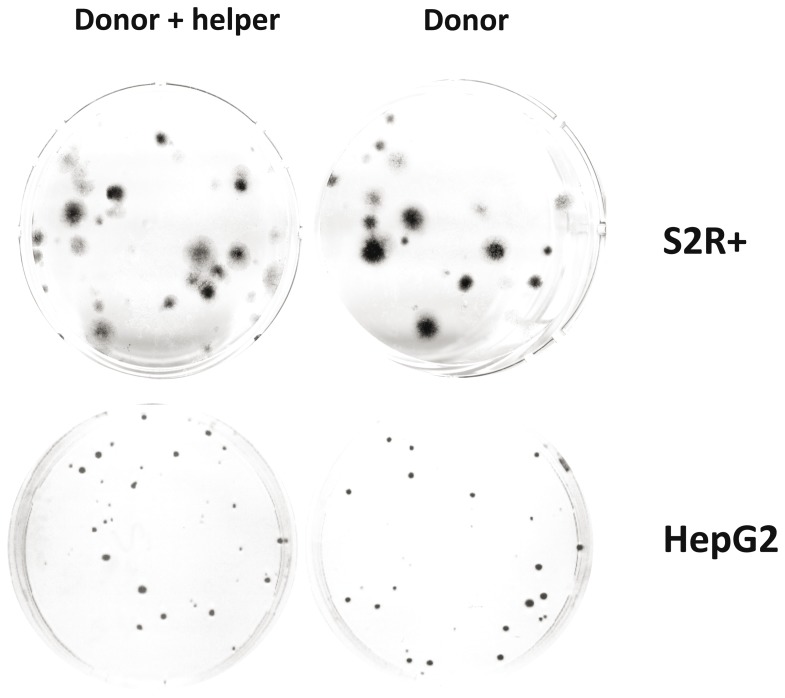
Transposition assay. Resistant colonies resulting from the transfection of Drosophila S2R+ cells (top) and human HepG2 cells (bottom) with a mixture of donor plasmid and helper plasmid or with the donor plasmid alone.

We have tested the transposition activity of *Bari1* using classic transposition assay. This is a simple assay aimed to demonstrate the activity of both isolated and reconstructed transposons; the assay is usually performed in heterologous cellular systems in order to bypass repressive circuitry acting on the original cellular environment from which the element has been isolated. On the other hand, testing transposition in homologous cellular systems ensures that necessary and species-specific co-factors are present. For these reasons, we have tested *Bari1* transposition in cultured human HepG2 or Drosophila S2R+ cells after co-transfection of a plasmid carrying a marked transposon containing a neomycin or blasticidin antibiotic resistance gene plus a helper plasmid expressing the *Bari1* transposase. As shown in figure 5, there was no increase in resistant foci with respect to the controls, indicating lack of transposition in either system assayed.

Similarly, a transposon excision assay based on PCR amplification of the “empty” donor plasmid molecules in cultured cells transfected as described above failed to detect transposon excision (data not shown).

#### Analysis of *Bari1* transcripts in the hsp83^scratch^ mutant

Thereafter, we asked if *Bari1* transposition could be affected by mechanisms acting at the transcriptional or post-transcriptional level. To overcome any possible repressive epigenetic mechanism acting on *Bari1* [24] [39] [40], we analyzed transcripts in homozygous hsp83^scratch^ *D. melanogaster* mutants.

**Figure 6:**
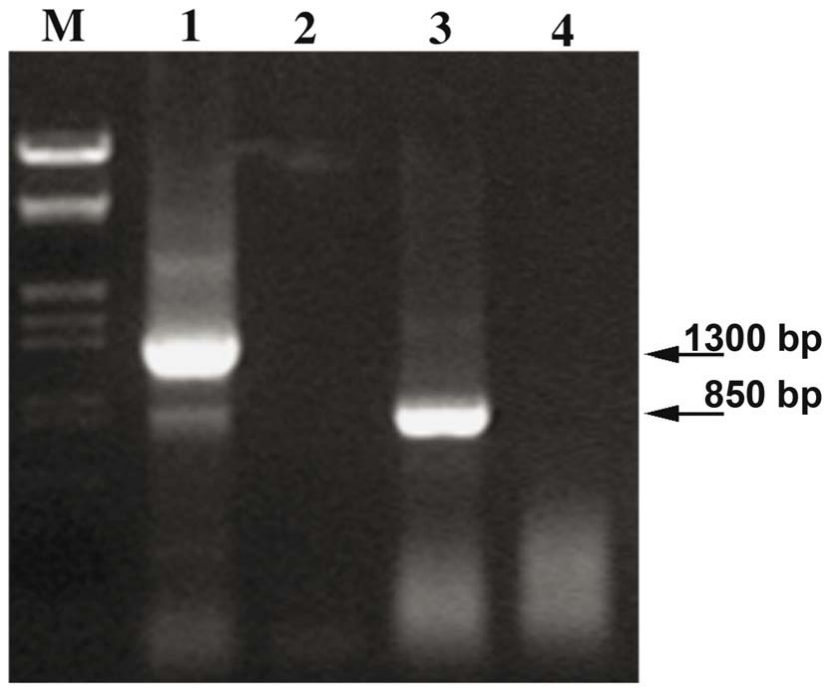
*Bari1* transcriptional analyses in the Drosophila hsp83^scratch^ strain. Results of RT-PCR performed on ovaries (lanes 1 and 2) and testes (lanes 3 and 4) of homozygous hsp83^scratch^ mutant (lanes 1 and 3) or wild type flies (lanes 2 and 4). M: **λ**/Eco-Hind molecular weight marker. Arrows indicates the approximate molecular weight of the amplified cDNAs.

As previously reported, in the hsp83^scratch^ mutant there is a strong germ line deregulation of several mobile genetic elements, including *Bari1* [24]. We analyzed by RT-PCR the *Bari1* transcripts from hsp83^scratch^ ovaries and testes, tissues where instability of the mobile genetic elements is known to occur. In contrast to the ovaries, where only a full-length 1,396 bp long cDNA was found, in the testes an 882 bp long cDNA was present (figure 6, lanes 1 and 3). No transcripts were detected in the somatic tissues (carcasses) of the hsp83^scratch^ strain (VS and MPB personal communication and unpublished) indicating transcriptional repression in somatic tissues *in vivo*.

**Figure 7:**
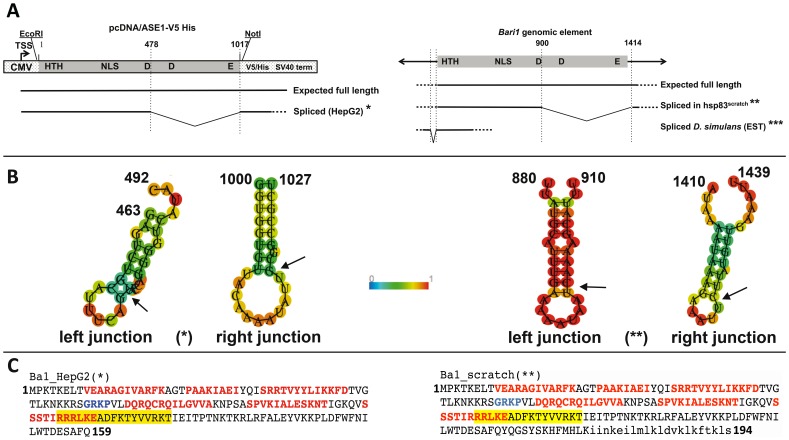
Structural analysis of the transcripts. Panel A. Structural analyses of the transcripts isolated in HepG2 cells (*), in the testes of hsp83^scratch^ homozygous males (**) or identified *in silico* by *D. simulans* ESTs database (***). TSS: transcriptional start site. Positions are relative to the pcDNA/ASE1 plasmid sequence (left) assuming as position 1 the first nucleotide of the cloned fragment, or to the full-length element sequence (right). Panel B. Secondary structure prediction of the RNA sequences across the splicing sites of the HepG2-specific transcript marked with an asterisk (left) and the hsp83^scratch^ –specific transcript marked with two asterisks (right). Arrows indicate the splicing sites. Colored scale indicates base-pairing (unpairing) probabilities. Panel C. Putative protein products of the transcripts isolated in HepG2 cells (left) and in the testes of hsp83^scratch^ homozygous males (right). Color code and protein residues numbering are match those showed in figure 1. New amino acids added upon RNA processing are shown in italic.

The shorter transcript harbors a 514 bp internal deletion with respect to the full-length transcript (figure 7, panel A, right, and figure S2). Interestingly, this male specific mRNA potentially encodes a 194 aa protein entirely lacking the catalytic domain of the transposase (figure 7, panel C, right). On the other hand, no *Bari1* transcripts were detected in the gonads of a wild type *D. melanogaster* strain (figure 6, lanes 2 and 4) confirming that *Bari1* is transcriptionally repressed in wild type flies.

To exclude the possibility that an aberrant genomic copy carrying the same deletion could produce the observed testis-specific transcript, we have performed a PCR analysis on genomic DNA extracted from hsp83^scratch^ homozygous mutants using the same primers used for the RT PCR analysis. The results are compatible with the absence of defective copies of *Bari1* in the mutant genome hsp83^scratch^ (figure S3).

#### Analysis of *Bari1* transcripts in HepG2 and S2R+ cells

To further support the above results we have analyzed the transcripts arising upon transient overexpression of the *Bari1* transposase in HepG2 cells. Although these cells are of somatic origin, this experimental system mimics in part the hsp83^scratch^ mutant and, therefore, no piRNA-mediated epigenetic regulation is expected. Sequencing of cDNA clones revealed two transcripts of different size: one corresponding to the expected 1,2 Kb full-length *Bari1* transcript, the second to an unexpected shorter transcript. The shorter cDNA harbors a 540 bp deletion extending from position 478 (relative to the full-length coding region) to the NotI cloning site present in the pcDNA/ASE1 plasmid expressing the *Bari1* transposase (see figure 7, panel A, left, and figure S2). Also this mRNA isolated in HepG2 cells potentially encodes a 159 aa protein entirely lacking the catalytic domain of the transposase (figure 7, panel C, left).

A similar analysis was performed in S2R+ cells transfected with the pAC5/ASE1-V5His plasmid. In this case we failed to detect any processed cDNA, being able to detect a single cDNA, corresponding in size to the full-length transcript, even after additional cycles of nested amplification (data not shown).

The shorter transcripts reported above are unlikely to originate from spliceosomal-mediated splicing events, because canonical donor and acceptor consensus sites are absent in the corresponding positions of the full-length transcript (see figure S2). Also, no potential splicing sites are identified *in silico* by the NNSPLICE tool [41] (not shown). However, a careful analysis of the full-length *Bari1* transcript by the RNAfold program [42] revealed the presence of a stem-loop secondary structures at the regions encompassing the sequences removed in both shorter transcripts described above. Figure 7B shows these structures as predicted in the “unspliced” transcripts. It is worth noting that the left and the right junctions share the respective cleavage positions, the left cleavage occurring at the first bond in the stem and the cleavage at the right junction occurring at the first bond in the loop (see arrows in figure 7B).

#### Analysis of *Bari1* transcripts in related Drosophila species

We have mined the main ESTs databases in search of *Bari1* transcripts carrying deletions similar to those observed in the above described cDNA clones. No similar defective transcripts can be found in the FlyBase and NCBI ESTs databases, except for a *D. simulans* cDNA clone (GenBank: DK347895.1; cloneID = dsif17g09), which differs from the full-length transcript by a 66 bp deletion in the UTR region, but it is probably the product of a canonical splicing event (figure 7, panel A left and figure S2, second pairwise alignment). No *Bari1* genomic copy carrying this 66 bp deletion was detected by a BLAST search of the *D. simulans* genomic databases, thus excluding that it is the transcriptional product of a defective copy of the transposon.

On the basis of these results, we suggest that *Bari1* transcripts may undergo processing, thereby resulting in catalytically inactive transposase polypeptides.

## Discussion

The importance of transponsable elements as source of variability in the eukaryotic genome and their use in the development of novel integration tools (e.g. for gene therapy and functional genomics) dictates a better understanding of their biology. The *Tc1/mariner* superfamily of transposable elements has been extensively studied, particularly in order to explain the wide diffusion of its members in a variety of animal populations. At present, much detailed information is available about the key structural and functional features of many mobile elements of this superfamily, e.g. about the tempo and mode of their invasion of host species [43] [36], about the mechanisms underlying the specificity of the “cut and paste” reaction [44], about the host strategies that limit the harmful effect of their mobility within the host genome [45], and about their value as vectors in transgenesis experiments [46]. The *Bari* transposons, belonging to this remarkably family, are present in almost every species of *Drosophila* so far analyzed [17] [16]. With the aim of identifying and characterizing in depth the structural and functional features underlying the intriguingly successful diffusion of many members of the *Tc1/mariner* family throughout a wide range of host species, we started a detailed analysis of the *Bari1* transposition process. In this paper, we focus on three informative aspects of this process, i.e: 1) the cellular localization of the transposase; 2) the transposon-transposase physical interaction; 3) the possibility that a novel regulative post-transcriptional mechanism based on alternative splicing may, at least in some cell types and genetic backgrounds, repress the transposition of the *Bari1* elements.

### The *Bari1* Transposase Possesses a Functional NLS and is Capable of Binding the Terminal Ends of the Transposon

In order to achieve correct subcellular localization nuclear proteins contain NLSs [47], and this is also the case for the transposases encoded by highly active elements such as *Activator* and *Mu* of maize [48] [49], *Tag1* of *Arabidopsis* [50], *mariner* of *Drosophila* [51] and *BmTc1* of the silkworm [52].

The nuclear compartmentalization of the transposase expressed by *Bari1*, supposed to act on the chromosomal copies of the transponson, was never characterized before. In order to address this issue we transiently overexpressed *Bari1* transposase and performed immuno-localization experiments both in S2R+ and HepG2 cells. Our finding that *Bari1* transposase localized to the nucleus of both systems strongly suggested the presence of a functional NLS signal. Subsequent deletion analysis suggested that the NLS signal should be included within the first 158 amino acids of the protein, and multiple alignment analysis allowed us to locate this signal at positions 106–121 of the transposase (see figure 1).

Purified *Bari1* transposase specifically binds to the left and right TIR of the transposon. Our *in vitro* results show that the binding occurs at the three DR sequences occurring at the transposon termini [16]. However, the binding to the right transposon end appears to be significantly less efficient as compared to the left one (figure 3, panel A and B). This could be due either to the (quite low) sequence divergence between the DRs of the right and left termini, or to the much higher divergence of the intervening sequences that separate the DRs. Since recognition of both ends is the initial and crucial step in the transposition mechanism [53], the decreased binding affinity of one of the terminal sequences could significantly affect transposition efficiency.

*In vivo* experiments performed in yeast highlighted a strong binding of the transposase to two out the three DRs: the transposase/DR interaction resulted strong for the Lo and Li DRs but very weak for Lm. Similar data have been previously reported for the *Sleeping Beauty* transposon [13], whose middle (half) DR enhances, but is not essential for transposition, suggesting that the Lm repeat contributes to the protein-DNA interactions without a direct involvement.

### Is *Bari1* Transposition Controlled by Additional Post-transcripional Regulation Mechanisms?

Despite the correct sub-cellular localization of the transposase and its ability to bind both TIRs of the element, no transposition or excision of *Bari1* elements was detectable by classic assays performed on the cultured cell lines we utilized. Several explanations might be possible. First, as previously suggested [23], a poor catalytic activity of the transposase could lead to genomic mobility of the element only in rare instances. In this case, specific amino acid substitutions in the region encompassing the catalytic domain of the transposase could significantly improve the efficiency of the transposition process. Essentially, this was the strategy that allowed the birth of the engineered *Sleeping Beauty* element [10].

As mentioned above, *Bari1* has been shown to be an active transposon in *D. melanogaster*, albeit with a limited mobility [21] [23], suggesting the existence of control mechanisms that maintain a very low frequency of transposition.

Controlling mechanisms are not uncommon: the mobility of several transposable elements is regulated by host loci (e.g. *gypsy*-flamenco [54] [55], *ZAM*-COM [56]) or by self-encoded repressors (e.g. *P* element [53]). Unstable strains, in which an allelic form of a controlling locus allows a higher rate of transposition, are a further demonstration of the existence of control systems regulating genomic transposon mobility. An unstable strain, in which several transposable elements, including *Bari1*, are deregulated, has been recently described [24].

Previous studies have highlighted that *Bari1* is subjected to tight germline post-transcriptional regulation by the piRNA pathway [24] [39] [40]. Similarly, a regulation system based on the siRNA post-transcriptional gene silencing protects somatic cells against *Bari1* transposition in *Drosophila* [57]. In addition, the chromatin state of *Bari1* genomic copies is directly linked to the piRNA pathway in germline cells [39] and possibly to the siRNA pathway [58].

The results presented in this work suggest that at least one additional level of control may exist on *Bari1*. This evidence comes from transcript analysis of overexpressed *Bari1* transposase in HepG2 cells, which should lack both piRNA- and siRNA-based regulation on *Bari1* as well as epigenetic control at the chromatin level, being *Bari1* normally absent in the human genome. We have observed that, in this particular experimental condition, unusually processed *Bari1* transcripts can be detected, opening a suggestive scenario to a hitherto unreported mechanism relying on post-transcriptional modifications of *Bari1* transcripts. Very similar results were found after *Bari1* transcript analysis in the testes of a *Drosophila* unstable mutant strain in which the piRNA pathway has been disrupted and transcription of *Bari1* has been previously demonstrated. We hypothesize that the predicted stem-loop secondary structures at the splicing sites of *Bari1* full-length transcript (see Figure 7 panel B), which are in part similar to the secondary structures of natural targets of the so-called “non-conventional” splicing system.

It has been postulated before that secondary structure can be important in pre-mRNA processing, and this has been in some cases experimentally documented [59] [60].

Unconventional splicing is a special class of splicing events, which does not involve spliceosomes and occurs in the cytoplasm. The unconventional splicing system recognizes mRNA secondary structures at the boundaries of the intervening intron sequence and plays a key role in the Unfolded Protein Response (UPR) [61], as it leads to the production of spliced mRNAs which can be translated into functional transcription factors as the transcription factors XBIP [62] and HAC1 [63]. These transcription factors activate UPR in mammals and in yeast respectively. Dmel\Xbp1 has been identified as the *D. melanogaster* counterpart of the mammalian XBP1 protein, and its transcript is subject to unconventional splicing in the salivary glands of third instar larvae [64]. Upon accumulation of unfolded protein in the endoplasmic reticulum, XBP1 mRNA is processed to an active form by the endonuclease inositol-requiring enzyme 1, IRE1. The resulting loss of 26 nt (23 nucleotides in *D. melanogaster* [64]) from the spliced mRNA causes a frame-shift and produces the XBP1(S) isoform, which is the functionally active transcription factor.

An attractive speculation is that similar mechanism could be involved in the regulation of transposon mobility, although at present the IRE1 protein has not been shown to target transposon transcripts, and XBP1 (HAC1 in yeast) is the only known transcript processed by IRE1. However, little it is known about the endonucleolytic activity of IRE1 on other cellular mRNAs including transcripts of transposon origin.

The short unexpected transcripts that we have detected upon transient overexpression of the transposase gene might be simply explained as a side effect of IRE1 activity induced by the activation of the UPR pathway. In the testes of hsp83^scratch^ mutants this side effect could be enhanced, as a consequence of the concomitant deregulation of multiple families of transposable element [24]. Similarly, in the HepG2 human cells the *Bari1* transcript could be “intercepted” by IRE1 upon transient overexpression of the transposase gene, which has been tested under the transcriptional control of a strong promoter. We hypothesize that the protein products potentially encoded by the unusually spliced transcripts (see figure 7 panel C) could play a role in transposition suppression, acting as a dominant negative form of the transposase. In fact although these putative proteins lack the catalytic domains they could be still able to bind the transposon termini (i.e. contain the DNA binding domain) and, possibly, to form heterodimers with wild type transposase molecules.

It is worth noting that we have not detected processed transcripts in transfected S2R+ cell, and in the ovary of the hsp83^scratch^ mutant (figure 6), suggesting that we are facing a complicate, and multi-level, regulation issue. Interestingly, the finding that *Bari1* fails to transpose in S2R+ cells indicates that the inhibition occurs even in absence of transcript processing suggesting that other general mechanisms control *Bari1* activity (i.e. folding or the poor catalytic activity).

In conclusion, we have performed a functional analysis of the *Bari1* transposon and observed that, despite the nuclear localization of the transposase and its ability to bind the transposon terminal inverted repeats, *Bari1* has no detectable transposition activity. We speculate that post-transcriptional processing of mRNA could interfere with the transposition of *Bari1* elements, particularly in absence of primary (i.e. piRNA-mediated) host defense mechanisms. This regulatory system could involve canonical (spliceosomal-mediated) or unconventional (i.e. IRE1-mediated) splicing and cooperate with other already known or yet to be discovered regulatory circuits in controlling the genomic mobility of the elements of the *Bari1* transposon family, and possibly the mobility of elements of other families as well. Further studies are needed to demonstrate our hypothesis and to assess the existence of additional levels of regulation of a member of the Tc1-mariner superfamily.

## Materials and Methods

### Drosophila Stocks and Cell Cultures

Fly stocks were maintained on standard cornmeal-agar medium at 25°C.

The hsp83^scratch^ strain has been described by Specchia and co-authors [24].

S2R^+^ cells (DGRC, Bloomington, USA) were cultured in Schneider’s insect medium supplemented with 10% FBS, 1% penicillin/streptomycin, at 26°C.

HepG2 cells (ATTC, Manassas, USA) were grown in Dulbecco’s minimum essential medium supplemented with 10% FBS, 200 mM glutamine, 1% penicillin/streptomycin, and maintained at 37°C with 5% CO_2_.

For transposition and excision assays either blasticidin (25 µg/ml) or G418 (1 mg/ml) were added to the medium as selective agents depending on the cell type used.

### Transfection and Immuno-detection of Recombinant Proteins

One day prior to transfection cells were seeded and let grow into 6-wells plates containing sterile glass coverslips. Respectively 1×10^6^ and 5×10^5^ S2R^+^ and HepG2 cells were transfected with 1 µg of purified plasmids DNA using TransIt LT1 (Mirus).

For immunofluorescence staining, the cells attached to slides were washed with phosphate-buffered saline and fixed with 4% formaldehyde for 10 minutes at room temperature followed by three washes in PBS. Blocking was performed with a solution containing 10% fetal bovine serum and 0,5% of Triton X-100 for 30 minutes followed by two washes in PBS for 2 minutes each.

Cells were incubated with a dilution 1∶500 of V5 antibody (Invitrogen) conjugated with FITC fluorochrome for 2 hours. After three washes in PBS, the cells were stained with DAPI (4',6-diamidino-2-phenylindole) and mounted with anti-fade (DABCO).

Slides were imaged under an Olympus epifluorescence microscope equipped with a cooled CCD camera. At least 100 positive cells per slide were observed. Grey-scale images, obtained by separately recording FITC and DAPI fluorescence, were pseudo-colored and merged to obtain the final image using Adobe Photoshop software.

### Expression Plasmids Construction

A PCR-based strategy was used to clone the transposase ORF, and the derivatives amino and carboxyl terminal fragments into the pAC5.1/V5-His vector (Invitrogen).

Bari1_UP/Bari1_Low, Bari1_UP/Bari1_N-Ter Low, Bari1_C-Ter Up/Bari1_Low respectively were used to amplify DNA sequences encoding the full length *Bari1* transposase, its NH-terminal and its COOH-terminal fragments.

The PCR products were digested with EcoRI and NotI restriction enzymes and cloned into the pAc5.1 V5-His C vector in-frame with and upstream the V5-His tag coding sequence of the plasmid. The fusion constructs were sub-cloned in pcDNA3.1 (Invitrogen) using EcoRI and BamHI. All plasmids were sequence-verified.

Cloning in the expression plasmid pET100/D-TOPO was performed using Ba381-topo-U/Ba1398L and Ba802U/Ba1398L to obtain the pET/T16 and pET/C9 respectively.

### Recombinant Protein Expression and Purification

Induction of the T16 protein was obtained in E. coli strain BL21(DE3) (Novagen) by the addition of 1 mM IPTG at 0.5 OD600 and continued for 2.5 hr at 37°C. Cells were sonicated in 25 mM HEPES (pH 7.5), 1 M NaCl, 15% glycerol, 0.25% Tween 20, 2 mM b-mercaptoethanol, 1 mM PMSF, and 10 mM imidazole (pH 8.0) was added to the soluble fraction before it was mixed with Ni-NTA resin (Qiagen) according to the recommendations of the manufacturer. The resin was washed with sonication buffer containing 30% glycerol and 50 mM imidazole; bounded proteins were eluted with sonication buffer containing 300 mM imidazole and dialyzed overnight against sonication buffer without imidazole.

### Electrophoretic Mobility Shift Assay (EMSA)

The fragments used in EMSA assays were obtained by amplification using the following primer combinations.

Ba_EW4/Ba_292L to obtain the full-length left TIR (figure 3 panel A, C). Ba_1422U/Ba_EW5 to obtain the full-length right TIR fragment (figure 3 panel B). Ba_A/Ba_292L to obtain the fragment depicted in figure 3 panel E. Ba_EW4/Ba_F to obtain the fragment depicted in figure 3 panel D. Ba_A/IR_Ba204 to obtain the fragment depicted in figure 3 panel F.

Fragments were cloned in the pGEMT-easy vector (Promega) and subsequently released by double digestion to obtain fragments with the protruding ends necessary for end labeling.

The fragments tested were end-labeled by mean of a filling-in reaction, using [**α** ^32^P]dATP and the Klenow fragment. Nucleoprotein complexes were formed in 25 mM Hepes pH 7.6, 1 mM EDTA pH 8, 50 mM NaCl, 1 mM DTT, 0.1 mg BSA, 2.5 mM Spermidin, 10% Glycerol, 0.1 mg poly [dI][dC] in a total volume of 20 **µ**l. Reactions contained 1 ng labeled probe, and 1.5 ng of purified transposase. After 20 min incubation on ice, 5 **µ**l of loading dye containing 50% glycerol and bromophenol blue was added and the samples loaded onto a 4% polyacrylamide gel in 0.25X TBE buffer.

### Yeast Methods

The YM4271 yeast strain was grown in SD medium supplemented with aminoacid, which complement auxotrophies.

Yeast transformation was performed using the TRAFO methods described in [65].

One-hybrid experiments were essentially carried out following the MatchMaker One Hybrid System manual (Clontech). Briefly, Synthetic oligonucleotides (see table S1) containing three tandem repeats of the outer, inner, or middle DRs were designed in order to create double stranded oligonucleotides with protruding ends compatible with the EcoRI and SalI sites of the pLacZi plasmid. Annealing was performed for the following oligonucleotides couples: Z3Lo+/Z3Lo−; Z3Lm+/Z3Lm−; Z3Li+/Z3Li− to obtain the pLaczi-3Lo pLaczi-3Lm pLaczi-3Lm plasmids respectively. Annealed oligonucleotides were cloned into the pLacZi plasmid vector. Similarly, the entire left TIR of *Bari1* was amplified with the Ba_EW4/Ba_292L oligonucleotides and cloned into pLacZi. These plasmids were linearized and independently transformed into the *S. cerevisiae* YM4271 strain in order to obtain integration at the URA locus. Integrants were selected on SD agar plates lacking uracil.

The background expression level of the reporter system was determined by a standard **β**-galactosidase assay. Colonies were transferred to Whatman filter paper discs and lysed with liquid nitrogen. Filters were then exposed to Z-buffer (Na2HPO4.7H2O 60 mM, NaH2PO4.H2O 40 mM, KCl 10 mM, MgSO4 1 mM, **β**–mercaptoethanol 50 mM, pH 7) containing X-gal (5-bromo-4-chloro-indolyl-β-D-galactopyranoside 0,33 mg/ml). Only clones without LacZ basal expression in 8 hours were selected for further analyses.

The selected positive colonies were then transformed with a plasmid expressing the prey protein, obtained by cloning the sequence encoding either the full-length transposase or the C terminal portion into the pACT2 vector into the EcoRI and XhoI sites and in frame with the GAL4-AD. **β**-galactosidase activity was assayed for 6 hours.

Oligonucleotides used for the transposase gene amplification were Ba381U_pACT2/Ba1381L_pACT2 and Ba805U_pACT2/Ba1381L_pACT2 for the full-length and the carboxyl terminal portion of the transposase respectively.

All recombinant plasmids obtained were sequence-verified.

### Transcriptional Analysis

RNA was extracted with TRIZOL. Cultured cells were directly processed after two washes in PBS 1X. Quantitation and estimation of RNA purity were performed using a NanoDrop spectrophotometer.

1 **µ**g RNA was converted to cDNA using the QIAQuick reverse transcription kit and following the manufacturer’s instruction. RNA samples from transfected HepG2 cells were amplified with the *Bari*1 UP/V5 Rev primer primers, while RNA samples from hsp83^scratch^ were amplified with the BaintB_UP/BaintB_low primers.

### Transposition and Excision Assays

Transposition assays were performed as described in [10].

Donor plasmids were constructed starting from the p28/47D plasmid clone containing a *Bari1* element with the flanking sequences from the 47D region of the polytene chromosomes. Either the blasticidin resistance cassette or the neomycin resistance cassette were amplified from the pCMV-beta and from the pCoBlast plasmids respectively and inserted into the KpnI site of *Bari1*. Helper plasmids were the same used for the subcellular localization of the transposase.

Cells were co-transfected with a donor plasmid and a helper plasmid expressing the full-length transposase. Two days after the transfection the medium was supplemented with the selective agent (blasticidin or G418) and cultured for two to three weeks.

For the excision assay, cells were co-transfected as described above and two to five days after transfection plasmid DNA was extracted from the transfected cells using the modified Hirt method [66]. Excision_FOR/excision_REV and M13for/M13rev oligonucleotides were used to detect “empty” donor plasmid molecules.

### *In silico* Methods

Pairwise alignments were performed using either the NCBI online tools or the LALIGN tool (http://embnet.vital-it.ch/software/LALIGN_form.html).

Multiple alignments were performed using the Multalin tool (http://multalin.toulouse.inra.fr/) [67]. Protein secondary structures predictions were performed using the PhD secondary structure prediction method (http://npsa-pbil.ibcp.fr/cgi-bin/npsa_automat.pl?page=/NPSA/npsa_phd.html) [68]. Sequences used for construction of the multiple alignment in figure 1 were retrieved from the Repbase database (www.girinst.org) [69].

NLS predictions were performed using the PSORT program (http://psort.hgc.jp/) [70].

RNA secondary structures were predicted using the RNAfold web service tool (http://rna.tbi.univie.ac.at/cgi-bin/RNAfold.cgi).

## Supporting Information

Figure S1 **Purification of the** ***Bari1*** **transposase protein.** Panel A. PAGE of several protein extract from different purification steps of the T16 protein. BL21: total BL21 cell lysate not induced; T16: total cell lysate from transformed BL21 cells without IPTG induction; T16*: total cell lysate from transformed BL21 cells after induction with 1 mM IPTG 37°C; T16*S: soluble fraction from T16*. The red arrow indicates the induced protein; T16*P: insoluble fraction from T16*; M: molecular weight marker. The 50 and 40 KD bands of the marker are indicated. Panel B. Western blotting with the anti 6HIS/GLY antibody specifically recognizes the induced T16 protein. Panel C. PAGE of several protein extract from different purification steps of the C9 protein. M: molecular weight marker (the 30, 25 and 20 KD bands are indicated); BL21-NI: total BL21 cell lysate not induced; BL21-I: total cell lysate after induction with 1 mM IPTG 37°C; C9-NI: total cell lysate from transformed BL21 cells without IPTG induction; C9-I: total cell lysate from transformed BL21 cells after induction with 1 mM IPTG 37°C. The red arrow indicates the induced protein.
(PPTX)

Figure S2 **Alignment of the processed transcripts identified in this study to the reference sequences.**
(DOCX)

Figure S3 **The genome of hsp83^scratch^ mutant does not contain defective copies of** ***Bari1*** **.** M – **λ**/Eco-Hind molecular weight marker; Lane 1 - PCR product from Oregon-R DNA; Lane 2 - PCR product from hsp83^scratch^ homozygous flies. Arrows indicates fragments of the expected size (about 1200 bp). Primers used are the same used in the RT-PCR experiments and described in the Methods section.
(TIF)

Table S1 **List of the oligonucleotides used in this study.**
(DOCX)
