# Supplementary material for: Functional Characterization of the Bari1 Transposition System
Source: PLoS One. 2013 Nov 14;8(11):e79385. doi: 10.1371/journal.pone.0079385 (PMC3828361; doi:10.1371/journal.pone.0079385)
Supplement: Figure S2 — Alignment of the processed transcripts identified in this study to the reference sequences. (DOCX) [file pone.0079385.s002.docx]

**1) Partial global sequence alignment between the transcript in transfected HepG2 cells (top) vs pcDNA/ASE1 plasmid (bottom). Intron is in red bold fonts. Note the absence of the consensus of splicing at the intron’s boundaries. The NotI cloning site is highlighted in yellow.**

10 20 30 40 50 60

ASE1-s TGATAGAATTTAAACATGCCCAAAACAAAAGAGTTAACAGTTGAGGCCCGGGCTGGTATT

:::::::::::::::::::::::::::::::::::::::::::::

pcDNA/ ---------------ATGCCCAAAACAAAAGAGTTAACAGTTGAGGCCCGGGCTGGTATT

10 20 30 40

70 80 90 100 110 120

ASE1-s GTTGCTAGGTTTAAAGCCGGTACACCTGCGGCCAAAATAGCTGAAATATATCAAATTTCG

::::::::::::::::::::::::::::::::::::::::::::::::::::::::::::

pcDNA/ GTTGCTAGGTTTAAAGCCGGTACACCTGCGGCCAAAATAGCTGAAATATATCAAATTTCG

50 60 70 80 90 100

130 140 150 160 170 180

ASE1-s CGTAGAACTGTCTACTACTTAATAAAAAAGTTTGATACAGTTGGCACATTAAAAAATAAA

::::::::::::::::::::::::::::::::::::::::::::::::::::::::::::

pcDNA/ CGTAGAACTGTCTACTACTTAATAAAAAAGTTTGATACAGTTGGCACATTAAAAAATAAA

110 120 130 140 150 160

190 200 210 220 230 240

ASE1-s AAAAGATCAGGCCGAAAACCTGTGCTGGACCAAAGGCAATGCAGGCAAATACTTGGAGTT

::::::::::::::::::::::::::::::::::::::::::::::::::::::::::::

pcDNA/ AAAAGATCAGGCCGAAAACCTGTGCTGGACCAAAGGCAATGCAGGCAAATACTTGGAGTT

170 180 190 200 210 220

250 260 270 280 290 300

ASE1-s GTGGCGAAGAATCCTAGTGCCAGTCCGGTAAAAATTGCCTTAGAATCAAAAAATACAATT

::::::::::::::::::::::::::::::::::::::::::::::::::::::::::::

pcDNA/ GTGGCGAAGAATCCTAGTGCCAGTCCGGTAAAAATTGCCTTAGAATCAAAAAATACAATT

230 240 250 260 270 280

310 320 330 340 350 360

ASE1-s GGCAAACAAGTTAGTAGTTCTACAATTCGTCGCAGGCTAAAAGAAGCTGATTTTAAGACA

::::::::::::::::::::::::::::::::::::::::::::::::::::::::::::

pcDNA/ GGCAAACAAGTTAGTAGTTCTACAATTCGTCGCAGGCTAAAAGAAGCTGATTTTAAGACA

290 300 310 320 330 340

370 380 390 400 410 420

ASE1-s TACGTTGTTCGCAAAACGATTGAGATCACACCAACCAACAAAACAAAACGTCTTCGATTT

::::::::::::::::::::::::::::::::::::::::::::::::::::::::::::

pcDNA/ TACGTTGTTCGCAAAACGATTGAGATCACACCAACCAACAAAACAAAACGTCTTCGATTT

350 360 370 380 390 400

430 440 450 460 470 480

ASE1-s GCGTTGGAATATGTTAAGAAGCCTCTTGACTTTTGGTTTAATATTTTATGGACTGATGAG

::::::::::::::::::::::::::::::::::::::::::::::::::::::::::::

pcDNA/ GCGTTGGAATATGTTAAGAAGCCTCTTGACTTTTGGTTTAATATTTTATGGACTGATGAG

410 420 430 440 450 460

490

ASE1-s TCTGCATTTCAG------------------------------------------------

::::::::::::

pcDNA/ TCTGCATTTCAG**TACCAGGGGTCATACAGCAAGCATTTTATGCATTTGAAAAATAATCAA**

470 480 490 500 510 520

ASE1-s ------------------------------------------------------------

pcDNA/ **AAGCATTTGGCAGCCCAGCCAACCAATAGATTTGGTGGGGGCACAGTCATGTTTTGGGGA**

530 540 550 560 570 580

ASE1-s ------------------------------------------------------------

pcDNA/ **TGTCTTTCCTATTATGGATTCGGAGACTTGGTACCGATAGAAGGAACTTTAAATCAGAAC**

590 600 610 620 630 640

ASE1-s ------------------------------------------------------------

pcDNA/ **GGATACCTTCTTATCTTAAACAACCATGCTTTTACGTCTGGAAATAGACTTTTTCCAACT**

650 660 670 680 690 700

ASE1-s ------------------------------------------------------------

pcDNA/ **ACTGAATGGATTCTTCAGCAGGACAATGCTCCATGCCATAAGGGTAGGATACCAACAAAA**

710 720 730 740 750 760

ASE1-s ------------------------------------------------------------

pcDNA/ **TTTTTAAACGACCTTAATCTGGCGGTTCTTCCGTGGCCCCCCCAAAGCCCAGACCTTAAT**

770 780 790 800 810 820

ASE1-s ------------------------------------------------------------

pcDNA/ **ATCATTGAAAACGTTTGGGCTTTTATTAAAAACCAACGAACTATTGATAAAAATAGAAAA**

830 840 850 860 870 880

ASE1-s ------------------------------------------------------------

pcDNA/ **CGAGAGGGAGCCATCATTGAAATAGCGGAGATTTGGTCCAAATTGACATTAGAATTTGCA**

890 900 910 920 930 940

ASE1-s ------------------------------------------------------------

pcDNA/ **CAAACTTTGGTAAGGTCAATACCAAAAAGACTTCAAGCAGTTATTGATGCCAAAGGTGGT**

950 960 970 980 990 1000

500 510 520 530 540

ASE1-s ------------GCGGCCGCTCGAGGTCACCCATTCGAAGGTAAGCCTATCCCTAACCCT

::::::::::::::::::::::::::::::::::::::::::::::::

pcDNA/ **GTTACAAAATAT**GCGGCCGCTCGAGGTCACCCATTCGAAGGTAAGCCTATCCCTAACCCT

1010 1020 1030 1040 1050 1060

550 560 570 580 590 600

ASE1-s CTCCTCGGTCTCGATTCTACGCGTACCGGTCATCATCACCATCACCATTGAGTTTAAACC

::::::::::::::::::::::::::::::::::::::::::::::::::::::::::::

pcDNA/ CTCCTCGGTCTCGATTCTACGCGTACCGGTCATCATCACCATCACCATTGAGTTTAAACC

1070 1080 1090 1100 1110 1120

610 620

ASE1-s CGCTGATCAGCCTCGACTGTGCCTT

:::::::::::::::::::::::::

pcDNA/ CGCTGATCAGCCTCGACTGTGCCTT

1130 1140 1150

**2) Partial global sequence alignment between the transcript of Bari1 in D. simulans (top) vs Bari1 (bottom). Intron is in red bold fonts. Note the presence of the consensus of splicing at the intron’s boundaries.**

Partial global alignment Ba1_Ds EST/Bari1

Ba1_Ds ------------------------------------------------------------

Bari1 AGGCAAATGAAGAGATCTTTATCAGTTGTCAGAAGTATTTGCACACGGTTTCGTCGCATC

190 200 210 220 230 240

10 20 30 40

Ba1_Ds -----------------GCAATTTCTTCTTCAGTGATTGGTTTAGAGTGACAAGTGCCG-

::::::::::::::::::::::::::::::::::::::::::

Bari1 ACAATTATTTTCACAACGCAATTTCTTCTTCAGTGATTGGTTTAGAGTGACAAGTGCCG**G**

250 260 270 280 290 300

Ba1_Ds ------------------------------------------------------------

Bari1 **TTTGTTTGCTTAAATACATTTAAATTATTGAATAAAAATTAGATTTAATCATTTTCCTAT**

310 320 330 340 350 360

50 60 70 80 90

Ba1_Ds -----TTATTAAATAAAATGCCCAAAACAAAAGAGTTAACAGTTGAGGCCCGGGCTGGTA

:::::::::::::::::::::::::::::::::::::::::::::::::::::::

Bari1 **TACAG**TTATTAAATAAAATGCCCAAAACAAAAGAGTTAACAGTTGAGGCCCGGGCTGGTA

370 380 390 400 410 420

100 110 120 130 140 150

Ba1_Ds TTGTTGCTAGGTTTAAAGCCGGTACACCTGCGGCCAAAATAGCTGAAATATATCAAATTT

::::::::::::::::::::::::::::::::::::::::::::::::::::::::::::

Bari1 TTGTTGCTAGGTTTAAAGCCGGTACACCTGCGGCCAAAATAGCTGAAATATATCAAATTT

430 440 450 460 470 480

160 170 180 190 200 210

Ba1_Ds CGCGTAGAACTGTCTACTACTTAATAAAACAGTTTGATACAGTTGGCACATTAAAAAATA

::::::::::::::::::::::::::::: ::::::::::::::::::::::::::::::

Bari1 CGCGTAGAACTGTCTACTACTTAATAAAAAAGTTTGATACAGTTGGCACATTAAAAAATA

490 500 510 520 530 540

220 230 240 250 260 270

Ba1_Ds AAAAAAGATCAGGCCGAAAACCTGTGCTGGACCAAAGGCAATGCAGGCAAATACTTGGAG

::::::::::::::::::::::::::::::::::::::::::::::::::::::::::::

Bari1 AAAAAAGATCAGGCCGAAAACCTGTGCTGGACCAAAGGCAATGCAGGCAAATACTTGGAG

550 560 570 580 590 600

280 290 300 310 320 330

Ba1_Ds TTGTGGCGAAGAATCCTAGTGCCAGTCCGGTAAAAATTGCCTTAGAATCAAAAAATACAA

::::::::::::::::::::::::::::::::::::::::::::::::::::::::::::

Bari1 TTGTGGCGAAGAATCCTAGTGCCAGTCCGGTAAAAATTGCCTTAGAATCAAAAAATACAA

610 620 630 640 650 660

340 350 360 370

Ba1_Ds TTGGCAAACAAGTTAGTAGTTCTACAATTCGTCG--------------------------

::::::::::::::::::::::::::::::::::

Bari1 TTGGCAAACAAGTTAGTAGTTCTACAATTCGTCGCAGGCTAAAAGAAGCTGATTTTAAGA

670 680 690 700 710 720

Ba1_Ds ------------------------------------------------------------

Bari1 CATACGTTGTTCGCAAAACGATTGAGATCACACCAACCAACAAAACAAAACGTCTTCGAT

730 740 750 760 770 780

**3) Partial global sequence alignment between the transcript detected in the testes of hsp83^scratch^ mutants (top) vs Bari1 (bottom). Intron is in red bold fonts. Note the absence of the consensus of splicing at the intron’s boundaries.**

Ba1_Sc ------------------------------------------------------------

Bari1 CCTATGCAGAGTCAGATGAAAGAAGAATTGAAAAAATAACTGTTCCTATGCGCAAGGAAG

130 140 150 160 170 180

Ba1_Sc ------------------------------------------------------------

Bari1 AGGCAAATGAAGAGATCTTTATCAGTTGTCAGAAGTATTTGCACACGGTTTCGTCGCATC

190 200 210 220 230 240

10

Ba1_Sc -------------------------------------------AGAGTGACAAGTGCCGG

:::::::::::::::::

Bari1 ACAATTATTTTCACAACGCAATTTCTTCTTCAGTGATTGGTTTAGAGTGACAAGTGCCGG

250 260 270 280 290 300

20 30 40 50 60 70

Ba1_Sc TTTGTTTGCTTAAATACATTTAAATTATTGAATAAAAATTAGATTTAATCATTTTCCTAT

::::::::::::::::::::::::::::::::::::::::::::::::::::::::::::

Bari1 TTTGTTTGCTTAAATACATTTAAATTATTGAATAAAAATTAGATTTAATCATTTTCCTAT

310 320 330 340 350 360

80 90 100 110 120 130

Ba1_Sc TACAGTTATTAAATAAAATGCCCAAAACAAAAGAGTTAACAGTTGAGGCCCGGGCTGGTA

::::::::::::::::::::::::::::::::::::::::::::::::::::::::::::

Bari1 TACAGTTATTAAATAAAATGCCCAAAACAAAAGAGTTAACAGTTGAGGCCCGGGCTGGTA

370 380 390 400 410 420

140 150 160 170 180 190

Ba1_Sc TTGTTGCTAGGTTTAAAGCCGGTACACCTGCGGCCAAAATAGCTGAAATATATCAAATTT

::::::::::::::::::::::::::::::::::::::::::::::::::::::::::::

Bari1 TTGTTGCTAGGTTTAAAGCCGGTACACCTGCGGCCAAAATAGCTGAAATATATCAAATTT

430 440 450 460 470 480

200 210 220 230 240 250

Ba1_Sc CGCGTAGAACTGTCTACTACTTAATAAAAAAGTTTGATACAGTTGGCACATTAAAAAATA

::::::::::::::::::::::::::::::::::::::::::::::::::::::::::::

Bari1 CGCGTAGAACTGTCTACTACTTAATAAAAAAGTTTGATACAGTTGGCACATTAAAAAATA

490 500 510 520 530 540

260 270 280 290 300 310

Ba1_Sc AAAAAAGATCAGGCCGAAAACCTGTGCTGGACCAAAGGCAATGCAGGCAAATACTTGGAG

::::::::::::::::::::::::::::::::::::::::::::::::::::::::::::

Bari1 AAAAAAGATCAGGCCGAAAACCTGTGCTGGACCAAAGGCAATGCAGGCAAATACTTGGAG

550 560 570 580 590 600

320 330 340 350 360 370

Ba1_Sc TTGTGGCGAAGAATCCTAGTGCCAGTCCGGTAAAAATTGCCTTAGAATCAAAAAATACAA

::::::::::::::::::::::::::::::::::::::::::::::::::::::::::::

Bari1 TTGTGGCGAAGAATCCTAGTGCCAGTCCGGTAAAAATTGCCTTAGAATCAAAAAATACAA

610 620 630 640 650 660

380 390 400 410 420 430

Ba1_Sc TTGGCAAACAAGTTAGTAGTTCTACAATTCGTCGCAGGCTAAAAGAAGCTGATTTTAAGA

::::::::::::::::::::::::::::::::::::::::::::::::::::::::::::

Bari1 TTGGCAAACAAGTTAGTAGTTCTACAATTCGTCGCAGGCTAAAAGAAGCTGATTTTAAGA

670 680 690 700 710 720

440 450 460 470 480 490

Ba1_Sc CATACGTTGTTCGCAAAACGATTGAGATCACACCAACCAACAAAACAAAACGTCTTCGAT

::::::::::::::::::::::::::::::::::::::::::::::::::::::::::::

Bari1 CATACGTTGTTCGCAAAACGATTGAGATCACACCAACCAACAAAACAAAACGTCTTCGAT

730 740 750 760 770 780

500 510 520 530 540 550

Ba1_Sc TTGCGTTGGAATATGTTAAGAAGCCTCTTGACTTTTGGTTTAATATTTTATGGACTGATG

::::::::::::::::::::::::::::::::::::::::::::::::::::::::::::

Bari1 TTGCGTTGGAATATGTTAAGAAGCCTCTTGACTTTTGGTTTAATATTTTATGGACTGATG

790 800 810 820 830 840

560 570 580 590 600 610

Ba1_Sc AGTCTGCATTTCAGTACCAGGGGTCATACAGCAAGCATTTTATGCATTTGAAAA-TAAT-

:::::::::::::::::::::::::::::::::::::::::::::::::::::: ::::

Bari1 AGTCTGCATTTCAGTACCAGGGGTCATACAGCAAGCATTTTATGCATTTGAAAAATAAT**C**

850 860 870 880 890 900

Ba1_Sc ------------------------------------------------------------

Bari1 **AAAAGCATTTGGCAGCCCAGCCAACCAATAGATTTGGTGGGGGCACAGTCATGTTTTGGG**

910 920 930 940 950 960

Ba1_Sc ------------------------------------------------------------

Bari1 **GATGTCTTTCCTATTATGGATTCGGAGACTTGGTACCGATAGAAGGAACTTTAAATCAGA**

970 980 990 1000 1010 1020

Ba1_Sc ------------------------------------------------------------

Bari1 **ACGGATACCTTCTTATCTTAAACAACCATGCTTTTACGTCTGGAAATAGACTTTTTCCAA**

1030 1040 1050 1060 1070 1080

Ba1_Sc ------------------------------------------------------------

Bari1 **CTACTGAATGGATTCTTCAGCAGGACAATGCTCCATGCCATAAGGGTAGGATACCAACAA**

1090 1100 1110 1120 1130 1140

Ba1_Sc ------------------------------------------------------------

Bari1 **AATTTTTAAACGACCTTAATCTGGCGGTTCTTCCGTGGCCCCCCCAAAGCCCAGACCTTA**

1150 1160 1170 1180 1190 1200

Ba1_Sc ------------------------------------------------------------

Bari1 **ATATCATTGAAAACGTTTGGGCTTTTATTAAAAACCAACGAACTATTGATAAAAATAGAA**

1210 1220 1230 1240 1250 1260

Ba1_Sc ------------------------------------------------------------

Bari1 **AACGAGAGGGAGCCATCATTGAAATAGCGGAGATTTGGTCCAAATTGACATTAGAATTTG**

1270 1280 1290 1300 1310 1320

Ba1_Sc ------------------------------------------------------------

Bari1 **CACAAACTTTGGTAAGGTCAATACCAAAAAGACTTCAAGCAGTTATTGATGCCAAAGGTG**

1330 1340 1350 1360 1370 1380

620 630 640

Ba1_Sc --------------------------------AAATAAAGAAATTCTTATGTTGAAATTA

::::::::::::::::::::::::::::

Bari1 **GTGTTACAAAATATTAGTATTGTATTTATATA**AAATAAAGAAATTCTTATGTTGAAATTA

1390 1400 1410 1420 1430 1440

650 660 670 680 690 700

Ba1_Sc GATGTTAAGCTGAAATTTACTAAATTAAGTTGAGTGAAAATACTTTTGAAGCGCAATAAA

::::::::::::::::::::::::::::::::::::::::::::::::::::::::::::

Bari1 GATGTTAAGCTGAAATTTACTAAATTAAGTTGAGTGAAAATACTTTTGAAGCGCAATAAA

1450 1460 1470 1480 1490 1500

710 720 730 740 750 760

Ba1_Sc CATGTGAAAATACTATTGACAACTTGCATGCATATTTTCTTTTGCTTTAAGCTTTGTACT

::::::::::::::::::::::::::::::::::::::::::::::::::::::::::::

Bari1 CATGTGAAAATACTATTGACAACTTGCATGCATATTTTCTTTTGCTTTAAGCTTTGTACT

1510 1520 1530 1540 1550 1560

770 780 790 800 810 820

Ba1_Sc ATGAACCGTTATCTTTCGTATTTCTTTTCGACTACCTTCTGCATAGATCAAGCTAAGCGA

::::::::::::::::::::::::::::::::::::::::::::::::::::::::::::

Bari1 ATGAACCGTTATCTTTCGTATTTCTTTTCGACTACCTTCTGCATAGATCAAGCTAAGCGA

1570 1580 1590 1600 1610 1620

830 840 850 860 870 880

Ba1_Sc TAAGAACTATTTCAGGCAAATCGGACAACAACAAGAAGAAATATAACAAAAAGAAGTTG-

:::::::::::::::::::::::::::::::::::::::::::::::::::::::::::

Bari1 TAAGAACTATTTCAGGCAAATCGGACAACAACAAGAAGAAATATAACAAAAAGAAGTTGA

1630 1640 1650 1660 1670 1680

Ba1_Sc ----------------------------------------------

Bari1 AGTTTGCAAATATTGTGCGTTGTGAAAATACTTTTGACCACCTCTG
