# Supplementary figures and images for: Functional Characterization of the Bari1 Transposition System
Source: PLoS One. 2013 Nov 14;8(11):e79385. doi: 10.1371/journal.pone.0079385 (PMC3828361; doi:10.1371/journal.pone.0079385)

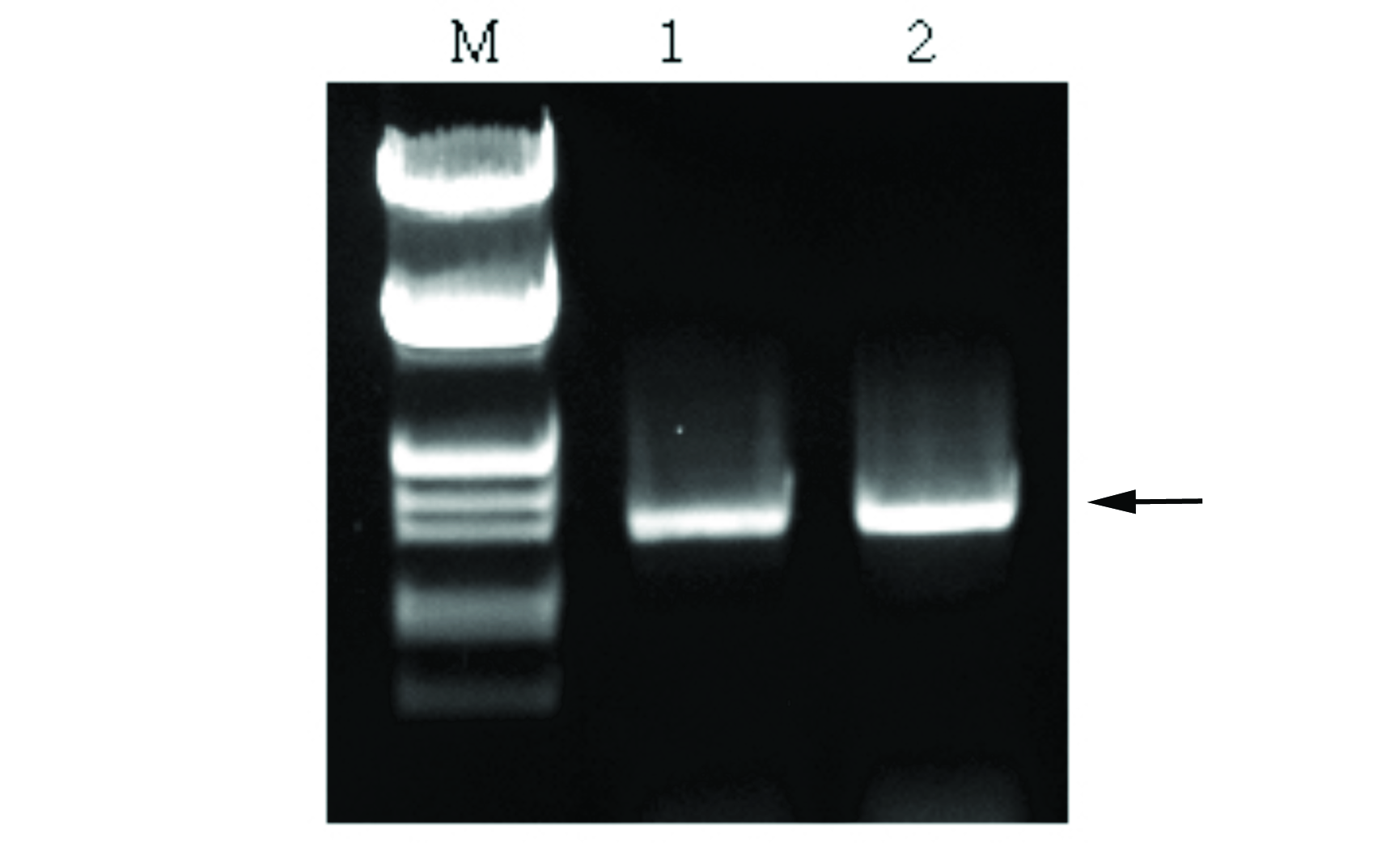

Supplement: Figure S3 — The genome of hsp83scratch mutant does not contain defective copies of Bari1 . M – λ/Eco-Hind molecular weight marker; Lane 1 - PCR product from Oregon-R DNA; Lane 2 - PCR product from hsp83scratch homozygous flies. Arrows indicates fragments of the expected size (about 1200 bp). Primers used are the same used in the RT-PCR experiments and described in the Methods section. (TIF) [file pone.0079385.s003.tif]
