# Supplementary material for: Functional Characterization of the Bari1 Transposition System
Source: PLoS One. 2013 Nov 14;8(11):e79385. doi: 10.1371/journal.pone.0079385 (PMC3828361; doi:10.1371/journal.pone.0079385)
Supplement: Table S1 — List of the oligonucleotides used in this study. (DOCX) [file pone.0079385.s004.docx]

| Oligo name | Sequence | Used for |
| --- | --- | --- |
| Z3Lo+  Z3Lo- | AATTCGTCAAAATTATTTTCACAGTCAAAATTATTTTCACAGTCAAAATTATTTTCACAG  TCGACTGTGAAAATAATTTTGACTGTGAAAATAATTTTGACTGTGAAAATAATTTTGACG | Cloning (plasmid obtained pLaczi-3Lo) |
| Z3Lm+  Z3Lm- | AATTCGTCAGAAGTATTTGCACAGTCAGAAGTATTTGCACAGTCAGAAGTATTTGCACAG  TCGACTGTGCAAATACTTCTGACTGTGCAAATACTTCTGACTGTGCAAATACTTCTGACG | Cloning (plasmid obtained pLaczi-3Lm |
| Z3Li+  Z3Li- | AATTCATCACAATTATTTTCACAATCACAATTATTTTCACAATCACAATTATTTTCACAG  TCGACTGTGAAAATAATTGTGATTGTGAAAATAATTGTGATTGTGAAAATAATTGTGATG | Cloning (plasmid obtained pLaczi-3Lo |
| Ba381U_pACT2  Ba1381L_pACT2 | TTTTGAATTCCCAAAACAAAAGAGTTAAC  TTTTCTCGAGCTAATATTTTGTAACACCA | Cloning(plasmid obtained pACT/ASE1 |
| Ba805U_pACT2  Ba1381L_pACT2 | TTTTGAATTCCTCTTGACTTTTGGTTTAA  TTTTCTCGAGCTAATATTTTGTAACACCA | Cloning(plasmid obtained pACT/ASE1 Δ1-143 |
| Bari1_UP | gatagaattcaaacatgcccaaaacaaaagagttaaca |  |
| V5_Rev | ACCGAGGAGAGGGTTAGGGAT | Sequencing; RT-PCR |
| Bgh_rev | TAGAAGGCACAGTCGAGG | Sequencing; RT-PCR |
| Ba381-topo-U | CACCCCCAAAACAAAAGAGTTAA | cloning |
| Ba802U | CACCAAGCCTCTTGACTTTTGGTT |  |
| Ba1398L | TTAACTAATATTTTGTAACACCACCTT |  |
| Bari1 Low | gataGCGGCCGcatattttgtaacaccacctttggca |  |
| Bari1_N-Ter Low | gataGCGGCCGcctgaaatgcagactcatcagtcca |  |
| Bari1_C-Ter Up | gataGAATTCaaacatgagtctgcatttcagtaccagg |  |
| BaintB_UP | AGAGTGACAAGTGCCGGTT | RT-PCR |
| BAintB_Low | CAACTTCTTTTTGTTATATTTCTTCTT | RT-PCR |
|  |  |  |
| Ba_A | AACAGTTGCTTGTGCAGCAAGTGGGG |  |
| Ba_F | AACCTATGCAGAGTCAGATGAAAGAA |  |
| Ba_292L | TGTCACTCTAAACCAATCACT |  |
| Ba_EW4 | CAGTCATGGTCAAAATTATTTTCACA |  |
| IR_Ba204 | AGAGGCAAATGAAGAGATCTTTAT |  |
| Ba_1422U | AATTCTTATGTTGAAATTAGATGTTA |  |
| Ba_EW5 | CAGAGGTGGTCAAAAGTATTTTCACA |  |
| Excision_FOR | GGCTGGCTTAACTATGCGGCATCAG |  |
| Excision_REV | GTCAGTGAGCGAGGAAGCGGAAGAG |  |
| M13for | GTTTTCCCAGTCACGAC |  |
| M13rev | CAGGAAACAGCTATGAC |  |
|  |  |  |
|  |  |  |
|  |  |  |
|  |  |  |
